# Understanding the Emerging Link Between Circadian Rhythm, Nrf2 Pathway, and Breast Cancer to Overcome Drug Resistance

**DOI:** 10.3389/fphar.2021.719631

**Published:** 2022-01-19

**Authors:** Supriya Bevinakoppamath, Shobha Chikkavaddaragudi Ramachandra, Anshu Kumar Yadav, Vijaya Basavaraj, Prashant Vishwanath, Akila Prashant

**Affiliations:** ^1^ Center of Excellence in Molecular Biology and Regenerative Medicine, Department of Biochemistry, JSS Medical College, JSS Academy of Higher Education and Research, Mysore, India; ^2^ Department of Pathology, JSS Medical College, JSS Academy of Higher Education and Research, Mysore, India; ^3^ Special Interest Group-Human Genomics and Rare Disorders, JSS Academy of Higher Education and Research, Mysore, India

**Keywords:** NRF2–KEAP1 pathway, breast cancer, BMAL-1, circadian rhythm, chemoresistance

## Abstract

The levels of different molecules in the cell are rhythmically cycled by the molecular clock present at the cellular level. The circadian rhythm is closely linked to the metabolic processes in the cells by an underlying mechanism whose intricacies need to be thoroughly investigated. Nevertheless, Nrf2 has been identified as an essential bridge between the circadian clock and cellular metabolism and is activated by the by-product of cellular metabolism like hydrogen peroxide. Once activated it binds to the specific DNA segments and increases the transcription of several genes that play a crucial role in the normal functioning of the cell. The central clock located in the suprachiasmatic nucleus of the anterior hypothalamus synchronizes the timekeeping in the peripheral tissues by integrating the light-dark input from the environment. Several studies have demonstrated the role of circadian rhythm as an effective tumor suppressor. Tumor development is triggered by the stimulation or disruption of signaling pathways at the cellular level as a result of the interaction between cells and environmental stimuli. Oxidative stress is one such external stimulus that disturbs the prooxidant/antioxidant equilibrium due to the loss of control over signaling pathways which destroy the bio-molecules. Altered Nrf2 expression and impaired redox balance are associated with various cancers suggesting that Nrf2 targeting may be used as a novel therapeutic approach for treating cancers. On the other hand, Nrf2 has also been shown to enhance the resistance of cancer cells to chemotherapeutic agents. We believe that maximum efficacy with minimum side effects for any particular therapy can be achieved if the treatment strategy regulates the circadian rhythm. In this review, we discuss the various molecular mechanisms interlinking the circadian rhythm with the Nrf2 pathway and contributing to breast cancer pathogenesis, we also talk about how these two pathways work in close association with the cell cycle which is another oscillatory system, and whether this interplay can be exploited to overcome drug resistance during chemotherapy.

## Introduction

**FIGURE 1:**
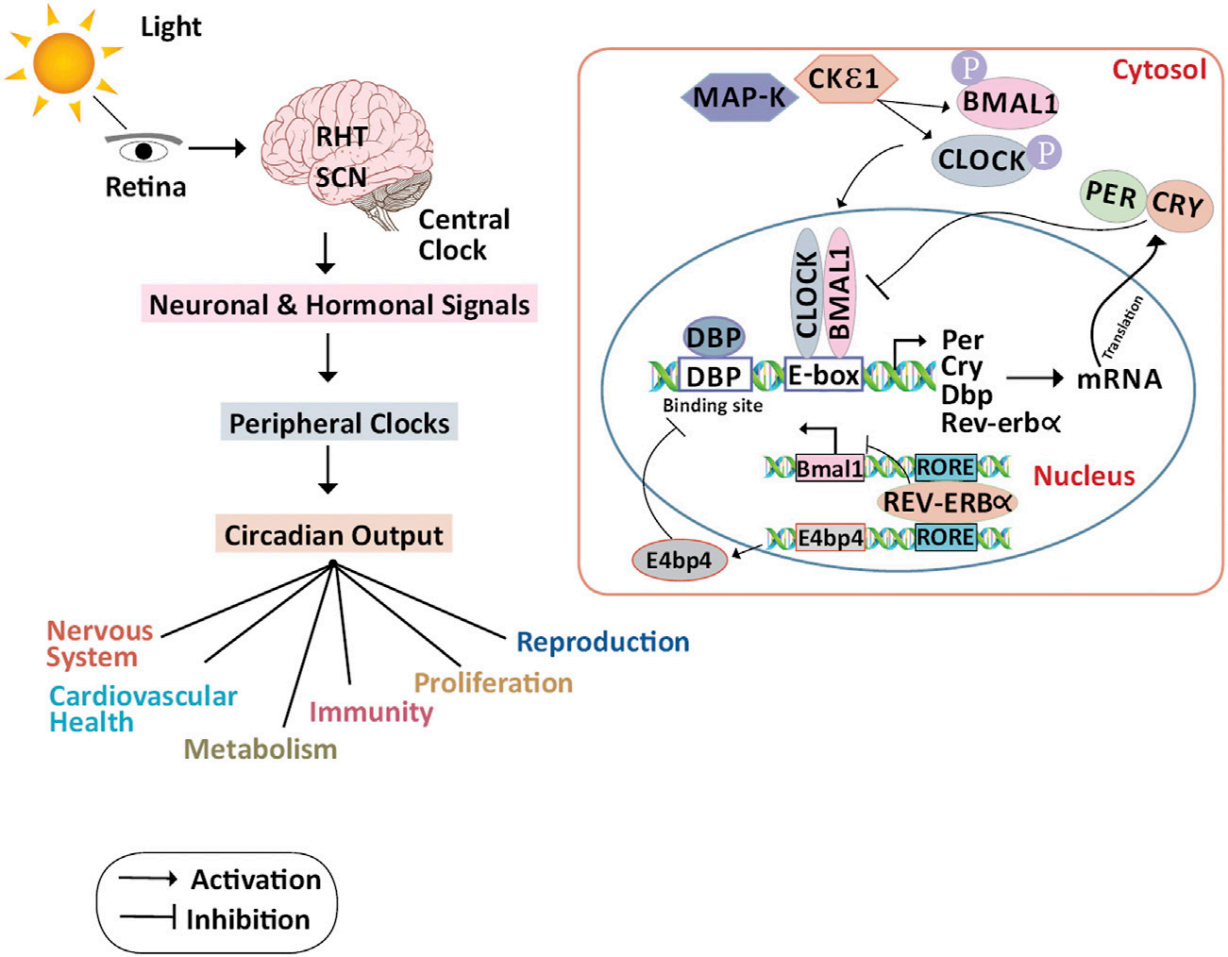
Molecular mechanism of the circadian clock and its physiological regulations: In the primary TTFL, as light strikes the retina, the impulses are routed by RHT in the SCN. The signals from the central clocks produced by SCN are transmitted to the peripheral organs as hormonal and neuronal signals, and as circadian outputs, several physiological functions are controlled. In the cytoplasm, CKIε and MAPK phosphorylate CLOCK and BMAL1. In the nucleus CLOCK/BMAL1 heterodimer binds to the Per, Cry, and other circadian genes through the E-box element present on the DNA and activates the transcription of these genes. PER and CRY proteins translocate to the cytoplasm and repress their transcription by interacting with CLOCK/BMAL1 heterodimer.DBP protein binds to the DBP binding site of the Per, Cry, and other circadian genes thus activating the transcription. The secondary TTFL is primarily regulated by transcriptional activation of the RORs and suppression of REV-ERBα. REV-ERBα binds to the ROR elements in the BMAL1 promoter, suppressing BMAL1 transcription, to guide the rhythmic oscillation of BMAL1. RORa and RORb which bind to the RORE, on the other hand, activate BMAL1 expression. E4bp4 activated by REV-ERBα represses the DBP. Abbreviations: BMAL1, Bone Muscle Arnt-like protein 1; CLOCK, Circadian Locomotor Output Cycles Kaput; CKIε, Casein Kinase ε; CRY, Cryptochrome; DBP, D-Box binding protein; E4bp4, E4 Binding Protein 4; MAPK, Mitogen-Activated Protein Kinase; PER, Period; REV-ERBα/NR1D1, Nuclear receptor subfamily 1, group D, member 1; RHT, Retinohypothalamic Tract; ROR, Retinoic acid receptor-related Orphan Receptor; RRE, ROR Responding Element; SCN, Suprachiasmatic Nucleus; TTFL, Transcription/Translational Feedback Loop.

The molecular circadian clock in mammals comprises input and output pathways with a central pacemaker located in the hypothalamic suprachiasmatic nucleus (SCN). The stimulus from the environment (such as light) is transmitted via the input pathway to the central pacemaker. Inside the SCN, several single-cell circadian oscillators are coordinated to provide regular circadian outputs. Signals from the SCN are converted into circadian oscillations through the output pathway. These oscillations work in tandem with peripheral organs and tissues to monitor physiological and behavioral functions (Welsh et al., 2004) (Figure 1).

Circadian oscillations are induced by two transcriptional/translational feedback loops (TTFLs) (Partch et al., 2014). In the primary TTFL, the activators of the core clock loop are Circadian Locomotor Output Cycles Kaput (CLOCK) (Gekakis, 1998) and Brain and Muscle Arnt-like protein 1 (BMAL1) (Honma et al., 1998), while the repressors are Period (PER1, PER2, and PER3) (Chen et al., 2009) and Cryptochrome (CRY1 and CRY2) (Lin and Farkas, 2018). CLOCK: BMAL1 heterodimer binds to the DNA promoter via the E-box and activates the transcription of PER, CRY, and other clock-controlled genes during the day. During the night, PER and CRY proteins translocate from the nucleus to the cytoplasm and dimerize, and this dimer interacts with the CLOCK: BMAL1 complex and inhibits their transcription at the E-box site (Thresher, 1998). PER and CRY are degraded by casein kinases (CKIδ and CKIε) and ubiquitin-dependent pathways thus releasing CLOCK: BMAL1 suppression (Busino et al., 2007; Lee et al., 2011; Siepka et al., 2007). The loop restarts with a 24-h cycle after PER and CRY have been degraded.

The secondary TTFL is regulated by retinoid-related orphan receptors (RORs a, b, and c) activation and REV-ERB suppression at the transcriptional level. To direct the rhythmic oscillation of BMAL1, REV-ERB binds to the ROR elements in its promoter, suppressing BMAL1 transcription. On the other hand, RORa and RORb allow the expression of BMAL1. Coordinated TTFLs with other kinases and phosphatases avoid environmental disturbances by regulating the oscillation time, phase and amplitude. This network also helps in the preservation of correct circadian timing, and its phase delay is adjusted to conform to local physiology (Brown et al., 2012). Disruption of the circadian clock function is linked to tumorigenesis in several studies with CLOCK and BMAL1 harboring tumor-suppressive roles. Also, there is evidence of endogenous production of reactive metabolites when the mammalian cells are exposed to variations in the circadian rhythm like night shift work (NSW). The transcriptional factor Nuclear factor erythroid 2-related factor 2 (Nrf2) protects the cells from these reactive metabolites by stimulating the expression of genes having antioxidant response element (ARE)-like sequences in their promoter. A limited search was conducted using PubMed using relevant keywords like Nrf2, breast neoplasm, circadian rhythm, CLOCK, BMAL, PER, CRY in the abstract and title. The identified terms used to search the literature, and the list and bibliography of articles were compiled from those found during the database search. Grey literature and articles that were not peer-reviewed were excluded. The period of the literature was no more than 10 years unless recent articles provided insufficient updates. This review took into account peer-reviewed articles that provided sufficient information about the relationship between circadian gene disruptions and breast cancer using human tissue samples, breast cancer cell lines, and mouse models. The disruption of core clock gene expression due to environmental cues and chemical inhibitors was the primary outcome of interest, with breast tumorigenesis being the secondary outcome. We discuss the various molecular mechanisms interlinking the circadian rhythm with the Nrf2 pathway and contributing to breast cancer pathogenesis and whether this interplay can be exploited to overcome drug resistance during chemotherapy.

## Circadian Rhythm Genes in Breast Cancer

From an epidemiological time point, circadian disruption has been associated with breast cancer (Richards and Gumz, 2013; Douma and Gumz, 2018; Thosar et al., 2018). It is evident from many studies that the circadian rhythm is an effective tumor suppressor. (Welz and Benitah, 2020; Verlande and Masri, 2019; Kiehn et al., 2017). Some of these studies have been summarised in Table 1. Especially Per2 and BMAL1 genes have been more pronounced as tumor suppressors (Curtis et al., 2014; Li et al., 2017; Verlande and Masri, 2019). Considering the role of Per2 in cellular and molecular levels, it is important to study the regulatory mechanisms in detail to understand its role in breast tumorigenesis. The expression of the Per2 gene in the early and mid of the light or dark phase determines the wax and wane in tumor size. Using therapy to target cancer cells when they are at their most proliferative stage could improve therapeutic tactics (Welsh et al., 2004). A transcription repressor Zinc finger protein (ZNF704) associated with the SIN3 transcription regulator family member A (SIN3A) complex is overexpressed and amplified in cancers including breast cancer. This promotes Epithelial-Mesenchymal Transition (EMT) by disrupting PER2 function and promoting early metastasis in breast cancer resulting in poor prognosis of the patients (Nakamura et al., 2001).

**TABLE 1:** Core clock genes and their association with breast cancer progression.

SI No	Gene	Role in breast cancer	Breast cancer models	Citation
1	Period 2 (PER2) down regulation	Increases the levels of Cyclin D1 and Cyclin E thus doubling the mammary tumor cell proliferation	Female C3HeB/FeJ (C3H) mice and Matured T-cell lymphoma (MTCL) cells	Yang et al. (2009)
2	Knockdown of Cryptochrome (Cry2)	Adverse effect on regulation of DNA repair mechanism and genome instability	MCF-7	Hoffman et al. (2010)
3	Per2 gene Suppression/depletion	Increased cell invasion	SKBR-3 and MDA-MB-231	Hwang-Verslues et al. (2013)
4	Expression of 17 clock components	Worse prognosis associated with loss of clock genes	Tumors from node- negative breast cancer patients	Cadenas et al. (2014)
5	Per2 silencing	Sensitivity towards cytotoxic effects of chemotherapeutic drug	MDA-MB-231	Mitchell and Engelbrecht, (2015)
6	ARNTL over expression	Resistance towards radiation	MCF-7	Mandl et al. (2015)
7	7 core clock transcription factors including Rev-Erba and Rorg	Increased oxidative stress and hepatic polyploidy	4T1-bearing breast cancer mice	Hojo et al. (2017)
8	Circadian expression of *Aldh3a1*	Circadian dynamics of the cancer stem cells are regulated by the circadian clock within the tumor microenvironment	4T1 mouse breast cancer cells	Matsunaga et al. (2018)
9	CLOCK, BMAL1, NPAS2, PER1, PER2, PER3 and CRY1, CRY2, TIMELESS, CSNK1E	Under-expression of PER1, PER2, PER3, CRY2 and over-expression of CLOCK, TIMELESS	Breast tumor tissue and adjacent normal tissue	Lesicka et al. (2018)
10	CLOCK, BMAL1, PERIOD (PER1, 2, 3), CRYPTOCHROME (CRY1, 2), and TIMELESS	Aberrant methylation patterns in circadian rhythm genes can be used as novel biomarkers	Breast tumor tissue and adjacent normal tissue	Lesicka et al. (2019)
11	Circadian oscillations of BMAL1 and PER2	Circadian rhythms are disrupted in high-grade tumors	MCF-7 and MDA-MB-231	Lellupitiyage Don et al. (2019)
12	Overexpression of BMAL1	Enhances cell invasion and metastasis	HEK-293T, MCF-7, T47D, ZR-75-30 and MDA-MB-231 cells	Wang et al. (2019)
13	Upregulation of TIMELESS (TIM)	Elevated cell proliferation and mitochondrial respiration	MCF7 and T47D	Zhang et al. (2020)

On the other hand, BMAL1 overexpression of Matrix Metalloprotein 9 (MMP9) up-regulates the expression of inflammatory factors that promotes breast cancer cell invasiveness and metastasis. Furthermore, studies reveal that recruiting CREB - Binding Protein (CBP) to boost the acetylating level of p65, further activating the NF-B signaling pathway in breast cancer, could be the underlying mechanism (Aschoff, 1965). A recent study on a primary tumor sample from a small population has underscored the association of single nucleotide polymorphisms (SNPs) in circadian genes associated with increased risk for breast carcinoma. SNPs of core circadian genes such as BMAL1 (rs2279287), CLOCK (rs12505266), CRY2 (rs10838524), PER1 (rs2735611), PER2 (rs934945, rs11894491) significantly influence the gene expressions based on their location on the gene. However, the risk of breast cancer depends upon the cancer subtype and the genotype. As a result, the finding of a link between cancer risk and polymorphism in a larger population could lead to novel therapeutic options (Tamura et al., 2008). Data from the previous studies have illustrated the role of TIMELESS (TIM) in breast cancer promotion. Elevated expression of TIM is involved in the Alkaline Ceramidase 2 (ACER2) metabolism essential for cancer cell survival and invasion by enhancing mitochondrial respiration (Patke et al., 2020). These shreds of evidence show the essential role of the circadian gene in tumorigenesis. Moreover, their genotyping and expression levels could serve as prognostic markers and may help to calculate disease-free survival. However, the underlying mechanisms are yet to be discovered even in-depth to improve the treatment.

## Circadian Rhythm and Physiological Functions

Animals exhibit cyclic fluctuations in behavior and physiology to accommodate daily repeated environmental transitions, which include notable compartmental conditions such as sleep-wake cycles as well as a slew of less noticeable oscillations in biochemical, physiological, endocrine, immunity, and cardiovascular functions. Entrainment is the process by which central and peripheral clocks are synchronized with the phase of external cues named zeitgebers. Light, eating patterns, temperature, physical activity, and mechanosensory activation are examples of zeitgebers that entrain circadian rhythms by operating either on the central pacemaker or directly in the peripheral tissues (Aschoff, 1965). A TTFL in animals which is at the center of a circadian cellular clock takes approximately 24 h, to monitor tissues and organs within the body via related downstream processes. Based on central and peripheral oscillations, mammal circadian physiology is sufficient to regulate the circadian cycle but it is supposed to provide a complex grid of humoral and neural pulses as well as body temperature rhythms that mediate circadian regulation downstream of the SCN (Patke et al., 2020).

Circadian clocks play a crucial role in many physiological processes, including cardiovascular, nervous, renal, immune, endocrine systems, etc (Richards and Gumz, 2013). Circadian clock dysregulation increases the probability of experiencing cardiovascular disease, with or without hypertension (Douma and Gumz, 2018). However, the underlying mechanisms related to cardiovascular diseases associated with circadian clock dysregulation are yet to be thoroughly investigated (Thosar et al., 2018). Circadian clocks are influenced by age and the aged SCN becomes less capable of collecting and transmitting signals to peripheral clocks (Welz and Benitah, 2020). Every cell in our body possesses a functional clock and disruption of which leads to the abnormal growth of the cells leading to various types of cancers (Verlande and Masri, 2019). Disruption of sleep, feeding activity, and Light At Night (LAN) has been found to affect circadian clock functioning, resulting in metabolic disorders. (Kiehn et al., 2017). It has been discovered that Parkinson’s symptoms are caused by the disruption in circadian rhythms, and circadian treatments are in high demand to treat the disease (Li et al., 2017). These pieces of evidence show that circadian rhythms are very important in maintaining homeostasis in our bodies. Dominant-negative mutant mice expressing the defective CLOCK/BMAL1 heterodimer show reduced expression of (C-X-C motif) ligand 1 (CXCL1) and interleukin-6 (IL-6). This indicates that the proper functioning of circadian clocks is also important in maintaining an adequate inflammatory response (Curtis et al., 2014). Melatonin is a hormone released by the endocrine system in response to the signals from SCN. It is an important key factor in regulating female fertility and pregnancy. During pregnancy, melatonin reduces oxidative stress and protects the baby from undergoing any harm by the ROS (Nakamura et al., 2001; Tamura et al., 2008; Yaw et al., 2021).

## Circadian Clock and NRF2/Antioxidant Responding Elements (ARE) Pathways

The mechanism of tumor development is controlled by the exposure of cells to external factors such as harmful or protective elements. Tumor development is triggered by the stimulation or disruption of signaling pathways at the cellular level as a result of the interaction between cells and environmental stimuli (Petric et al., 2015; Prahallad and Bernards, 2016; Zimta et al., 2019). Oxidative stress is one such external stimulus that disturbs the prooxidant/antioxidant equilibrium due to the loss of control over singling pathways which destroy the bio-molecules (Law and Sudweeks, 1975; Jones, 2006). Stress-related DNA adduct formation and aggregation as a result of incomplete DNA repair is an established reality as oxidative stress is elevated in cells. Antioxidant enzymes that sustain redox equilibrium in our body include peroxiredoxin (Prdx), superoxide dismutase (SOD), catalase (CAT), and glutathione peroxidase (GPx) (Barnes et al., 2018; Tascioglu Aliyev et al., 2021). Nrf2 is a transcription factor that belongs to the Cap “n” Collar (CNC) family and is encoded by the NFE2L2 gene (Moi et al., 1994). Its expression controls the expression of many cytoprotective genes implicated in antioxidant and anti-inflammatory responses (Tascioglu Aliyev et al., 2021).

The Nrf2/ARE pathway is regulated by the circadian clock and is essential for oxidative stress management in our body (Pekovic-Vaughan et al., 2014). Qian Sun has shown that the BMAL1 binds to the Nrf2/ARE promoter through the E-box element thus regulating the rhythmic activation of the Nrf2 transcription factor. When the expression of Nrf2/ARE is at zero circadian time point, expression of glutamate-cysteine ligase modifier (GCLM), NAD(P)H dehydrogenase [quinone] 1 (NQO1), and heme oxygenase 1 (HMOX1) proteins are weakened, resulting in severe oxidative injury (Tonelli et al., 2018; Sun et al., 2021). Nrf2 and its downstream antioxidant stress proteins are activated by BMAL1. To do so, BMAL1 binds to the PPAR promoter through the E-box element activates the transcription of ARE and other key antioxidant proteins in a circadian rhythm. The interaction of the circadian components withNrf2-mediated antioxidant,anti-apoptotic, Heat Shock Response (HSR), and NF-kappa-B-mediated pathways perform critical defensive roles in a variety of ROS-inducible illnesses, infections, and death (Tamaru et al., 2013). Research on human lens epithelial cells (LECs) discovered an age-dependent decrease in BMAL1-CLOCK and Nrf2-mediated antioxidant gene expression, as well as a gradual rise in ROS levels. Cells that overexpress BMAL1 showed increased, NQO1, SOD, Nrf2, Prdx6s mRNA, and protein levels, and this increased expression is closely linked to decreased ROS levels. BMAL1 resets the antioxidant pathway’s circadian cycle to maintain lens homeostasis (Chhunchha et al., 2020). These shreds of evidence reveal that the BMAL1 is not just limited to regulating circadian rhythm but also redox homeostasis (Stangherlin and Reddy, 2013). Nrf2 also plays a very important role in the innate immune system in limiting inflammation by suppressing ROS and repression of proinflammatory cytokines. As BMAL1 is the direct regulator of Nrf2, its deletion down-regulates the Nrf2 mediated antioxidant pathways, and there will be a significant increase in the production of IL-1β (Early et al., 2018). The loss of Nrf2 activity in mouse fibroblasts, hepatocytes, and liver resulted in altered circadian rhythms, implying that Nrf2 stoichiometry and/or timing of expression are essential for timekeeping in certain cells. In hepatocytes, activated Nrf2 binds to the unique enhancer regions of the central clock suppressor gene Cry2 and increases the expression of Cry2 while suppressing the CLOCK/BMAL1-regulated E-Box. These findings suggest that Nrf2 and the clock form an interconnected loop that incorporates cellular redox signals into tissue-specific circadian timekeeping (Wible et al., 2018). While several researchers have demonstrated the role of Nrf2 as a regulator of genes containing AREs, the effect of genetic Nrf2 gain-of-function on circadian rhythm or the circadian gene transcription has yet to be thoroughly investigated. The diurnal oscillation of intracellular redox potential is understood to connect metabolism and the circadian clock, but the mechanisms that underpin this are unclear.

## Disruption of Nrf2-Mediated Antioxidant Responses Resulting in Carcinogenesis

The internal metabolism and exposure to external stimuli generate reactive oxygen species (ROS) and reactive nitrogen species (RNS) constantly in the body. Physiologically, reactive oxidants produced act to regulate processes like division of the cell, inflammation, autophagy, etc (Finkel, 2011). Whereas under uncontrolled conditions, oxidative stress sets in, altering the functions of the cell and thus contributing to chronic disorders including cancer (Kensler et al., 2007; Ma, 2010). Moderate levels of ROS act as a signaling molecule, whereas uncontrolled levels cause DNA damage, thus a balance between synthesis and elimination of ROS should be maintained for redox homeostasis (Rhee, 2006). Most of the enzymes utilizing molecular oxygen as substrate synthesize ROS whereas, RNS is formed by the enzyme nitric oxide synthase that is later neutralized by antioxidants (Ma, 2013). Nrf2 is the master regulator of antioxidant responses and belongs to basic-region leucine zipper (bZIP) transcription factors together with NF-E2 p45, Nrf3, Nrf1 subunit (Tonelli et al., 2018). The modular protein, Nrf2 has Nrf2-ECH homology (Neh) domains ranging from Neh1–7.

**FIGURE 2:**
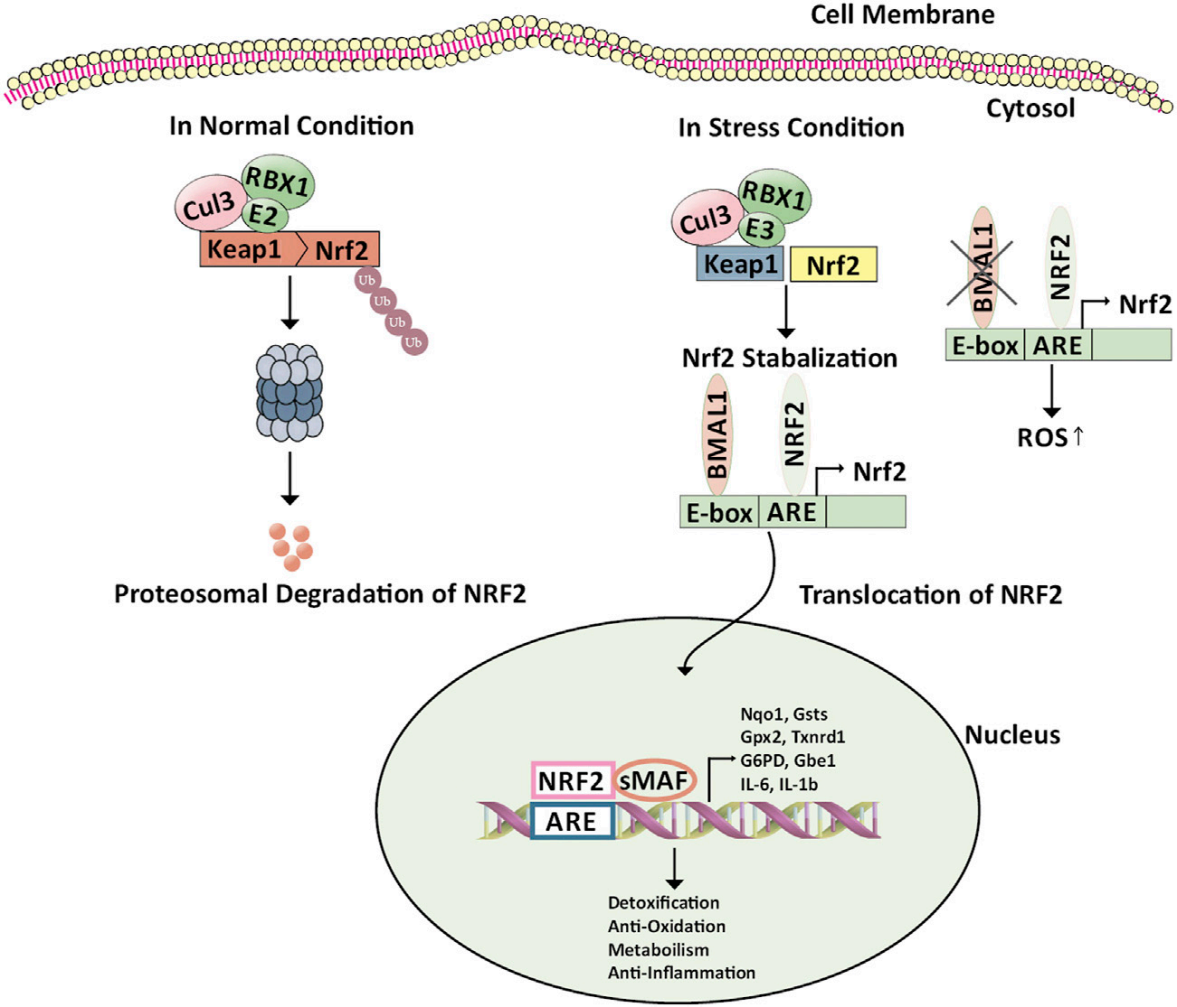
Regulation of Keap1/Nrf2/ARE pathway by BMAL1. Nrf2 is constitutively produced in the cell; however, in the absence of environmental stress, Nrf2 is sequestered in the cytoplasm by binding to an inhibitory protein, Keap1, which promotes continuous ubiquitinylation. Keap1 serves as a bridge between Nrf2 and the Cul3-Rbx1 E3 ubiquitin ligase. Cellular stress leads to modification of reactive cysteines within Keap1 that induces conformational changes resulting in Nrf2 stabilization. Here BMAL1 binds to the antioxidant response element through the E-box element present on the Nrf2 promoter leading to the transcription of Nrf2 and stabilization of the same. The NRF2 protein then translocates into the nucleus. There, it forms a heterodimer with other transcription regulators, such as small Maf proteins. The NRF2-sMaf complex binds to the ARE-containing genes in the nucleus involved in detoxification, anti-oxidation, anti-inflammation, and metabolism. The deficiency of BMAL1 disrupts the circadian rhythm of the Nrf2 pathway leading to cellular damage. Abbreviations: ARE, Antioxidant response element; BMAL1, Bone Muscle Arnt-like protein-1; Cul3-Rbx1 E3 ubiquitin ligase, Cullin 3-Ring box1 E3 ubiquitin ligase; E-box, Enhancer Box; G6pd, Glucose-6-phosphate Dehydrogenase; Gpx2, Glutathione Peroxidase 2; Gsts, Glutathione S-Transferase; IL-6, Interleukin-6; IL-1b, Interleukin 1 beta; Keap1, Kelch-like ECH-associated protein 1; NRF2, Nuclear factor erythroid 2-related factor 2; Nqo1, (NAD(P)H Quinone Dehydrogenase 1); sMaf, Small musculoaponeurotic fibrosarcoma; Txnrd1, Thioredoxin Reductase 1; Ub, Ubiquitin.

In normal conditions, Nrf2 is tightly bound to Keap1 and has a very short life span of 10–30 min. The expression of Nrf2 is regulated at the protein level (Figure 2). After translation, in the cytosol, the Keap1 homodimer attacks Nrf2 and facilitates binding of Cul3/Rbx1 E3 ubiquitin ligase complex, mediated by two Nrf2 sequences present in Neh2 domain leading to ubiquitylation of Nrf2. Next, Nrf2 is degraded rapidly by proteasome keeping low basal levels of Nrf2 (Eggler et al., 2009; Na and Surh, 2014; Fukutomi et al., 2014; Cullinan et al., 2004; Kobayashi et al., 2004; Nguyen et al., 2003). In the state of stress, ROS level rises, cysteine residues which are highly reactive in Keap1 are oxidized, as a result, Nrf2 disassociates from Keap1, and translocates into the nucleus. In the nucleus, Nrf2 heterodimers with a small musculoaponeurotic fibrosarcoma (sMAF) and bind to antioxidant response elements (AREs). Thus expression of genes is regulated in the promoter region which contains the MAF recognition elements (MARE) (Na and Surh, 2014; Moon and Giaccia, 2015). Nearly 250 genes, for example, NQO1, Glutathione (GSH) synthesis enzyme, Prdx, HMOX1, glutamate-cysteine ligase (GCL), are controlled by Nrf2 (Zimta et al., 2019). The Nrf2 attacks these metabolic enzymes and regulates redox homeostasis by detoxifying ROS and repairing oxidative damage. Thus, activation of Nrf2 can be considered a productive process to prevent cancer (Kwak and Kensler, 2010).

Different mechanisms activate Nrf2 in malignancy which disrupts the Keap1-Nrf2complex by epigenetic modifications, genetic mutations, hormonal activation, oncogenic signaling, stress signaling, RNA processing, etc (Jung et al., 2018). The mutation of Nrf2 and Keap1 alters their binding accounting for Nrf2 signaling overactivation (Hast et al., 2014). This has been seen in many cancers involving breast, prostate, gastric, liver, etc. The Nrf2 mutations are mostly found within the regions encoding the motifs, whereas the Keap1 mutations occur throughout the gene (Shibata et al., 2008; Jung et al., 2018). p62 also called sequestosome 1 (SQSTM1), is the Keap1-Nrf2 disruptor which directly binds to Keap1, competing with Nrf2 thus, bringing about autophagic degradation of Keap1. Studies have shown that there is an upregulation of p62 in hepatocellular carcinoma (Umemura et al., 2016). The other Keap1-Nrf2 disruptor dipeptidyl-peptidase 3 (DPP3) is over-expressed in estrogen receptor-positive breast cancer leading to poor prognosis (Lu et al., 2017). Thus Nrf2 over-activation is responsible for the enormous growth and proliferation capacity of the cancer cells.

The microRNA also regulates the Nrf2 signaling pathway in cancer. The redox miRs are the microRNAs that attack both Nrf2, Keap1, and a few other proteins which are intricated in the response of the cell against oxidative stress. These microRNAs may be either underexpressed which inhibits the progression of cancer or maybe overexpressed which enhances cancer progression (Zimta et al., 2019). Nrf2 silencing in breast cancer leads to overexpression of miR-181c and NF-*k*B signaling pathways. Thus there is inhibition of cytochrome c oxidase production from mitochondria and consumption of oxygen (Jung et al., 2017). Nrf2 induces the transcription of microRNAs miR-365, 193b, 32, 1207, etc by binding to the encoding DNA sequences in the promoter region (Shah et al., 2013). This demonstrates the significant role, the Nrf2 signaling pathway plays in tumorigenesis and highlights the fact that indirect methods based on microRNAs can also be explored to target the Nrf2 pathway.

## Nrf2 Mediated Stabilization and Regulation of Circadian Clock

As mentioned previously, the exposure of mammalian tissues and cells to changes in the environment regularly produce ROS. For the normal functioning of the tissue and cells, effective scavenging of the synthesized ROS is essential. The circadian clock plays a vital role to maintain the ROS at normal levels and protect the tissues and cells from oxidative damage (Stangherlin and Reddy, 2013; Patel et al., 2014). The oxidative stress may be mastered by the coordination between the signaling of ROS by Circadian Clock-Associated 1 (CCA1) and circadian output. The circadian clock controls the production of ROS, its response, and its gene regulation transcriptionally. The ROS genes show the diurnal pattern of expression, which also affects the activity of the enzyme, catalase, and H_2_O_2_ production. Also, loss of CCA1 and Late Elongated Hypocotyl (LHY), the morning expressed transcription factors, alters the diurnal production of ROS and its scavenging. The circadian clock and the mutation of its components lead to alteration in response to oxidative stress (Lai et al., 2012).

The homeostasis of ROS is tightly regulated by antioxidant systems like Nrf2 transcriptional regulation which is also dependent on the circadian clock (Stangherlin and Reddy, 2013; Patel et al., 2014). The alteration of the glutathione-mediated Nrf2 pathway has a major role in the pathogenesis of cancer and several other diseases like pulmonary fibrosis (Pekovic-Vaughan et al., 2014). Evidence shows that the expression of Nrf2 is disrupted due to the ClockΔ19 mutation, resulting in low levels of reduced glutathione and high levels of oxidative damage. The BMAL1 and CLOCK regulating the transcription of Nrf2, pile up this protein in circadian form and operate the transcription of the foremost gene, engaged in the metabolism of glutathione. The Nrf2 exerts its activity by recruiting rhythmically to the AREs of the targeted genes, thus protecting against oxidative injury (Pekovic-Vaughan et al., 2014; Shim and Imaizumi, 2015). The CNC overexpression alters the synaptic mechanism which has a positive effect on the functions of neurons. Through the suppression of Keap1, the CNC inhibitor has a beneficial effect on synaptic function. The redox-sensitive behavior of sleep is impacted by the function of the synapse which is connected to diseases associated with circadian rhythm defects. Thus redox homeostasis is regulated through modulation of CNC/Keap1 signaling and regulates various aspects involving the nervous system like sleep behavior, aging, and function of the synapse (Spiers et al., 2019). The oxidative stress due to alteration in circadian rhythm initiates pro-inflammatory condition which may compromise the exchange between the key antioxidant (Nrf2) and inflammatory (NF-κB) pathways (Ramos-Tovar and Muriel, 2020; Frigato et al., 2020; Valacchi et al., 2017). A study to understand the relationship between the Nrf2, NF-κB pathways, and the circadian clock was performed by treating with lipopolysaccharide to induce chronic inflammation and analyzing both pathways in clock synchronized and desynchronized cells by exposing acutely to ozone. The result from this study suggested that circadian clock synchronized cells have a more effective and faster response to antioxidants and curb the damage of cells from chronic inflammatory conditions (Frigato et al., 2020). Another study was done using Nrf2/ARE pathway against the circadian clock by inducing oxidative stress by ischemic reperfusion of the renal system. They showed that the Nrf2/ARE pathways regulated by endogenous circadian genes are involved in protection against oxidative stress mechanisms. BMALI regulates the Nrf2 gene and the dysrhythmia of the antioxidant pathway alters the expression of antioxidant proteins that negotiate the periodic regulation to change the sensitivity to stress-induced by ischemic-reperfusion. Hence, it was concluded that the circadian clock controlling the circadian rhythm of the antioxidant pathway plays a major role in antioxidant stress regulation (Sun et al., 2021). Removal of BMAL1 leads to the altered activity of Nrf2 and leads to the buildup of ROS and IL-1β, the pro-inflammatory cytokine. Hence, the circadian clock controls the antioxidant role to maintain the IL-1β and transcriptional Nrf2 activity (Early et al., 2018).

## Role of the Biological Clock in the Regulation of Antioxidant Response

There are natural antioxidants which are produced endogenously through cellular metabolism and exogenously through diet to combat the deleterious effect of ROS and RNS formed. Mitochondrial DNA is more prone than nuclear DNA to damage caused by oxidative injury and mutation in it can cause alteration in the respiratory chain leading to loss of control over ROS production. The mitochondrial DNA is highly susceptible to change caused by oxidative damage for the reason that there is no protection from histones, intronless region, and high rate of transcription, and increased sensitivity to alteration in their coding region caused by oxidative damage (Dalle-Donne et al., 2003; Nita and Grzybowski, 2016). Melatonin, a ubiquitous substance that has antioxidant property is secreted from the pineal gland during the dark and regulates circadian rhythm, sleep pattern, anticancer activities, etc. It boosts the antioxidant activity of the cells by synthesizing antioxidant enzymes and also counteracts oxidative mitochondrial DNA damage. Decreased melatonin or altered rhythm of melatonin leads to increased oxidative stress and degenerative changes. This may be attributed to physiological age than chronological age (Barzilai et al., 2010; Ganguly et al., 2021). The circadian clock is one of the natural ways to coordinate the daily defense of antioxidants in rhythm. It regulates the enzyme expression, repairs the damage caused by ROS, and produces melatonin, thus maintaining homeostasis of ROS and defense activity of antioxidants daily (Patel et al., 2014). The synthesis of oxidants in a rhythmic manner facilitates activation of the antioxidant system in a rhythmic way to combat this oxidative stress. The detoxification of ROS is a multi-chain reaction, hence the upregulation of anyone enzyme is inadequate for organized and systematic detoxification and requires coordination with multiple enzymes for effective detoxification (Krishnan et al., 2008).

The core clock regulates the genes of redox hemostasis and instructs the cell to respond to oxidative damage. The *Per01* mutant flies showed altered circadian rhythm when compared to non-mutated flies and they had reduced life span, elevated oxidative damage, and degeneration of neurons (Krishnan et al., 2009; Krishnan et al., 2012). Oxidative stress may induce late-life cycler’s rhythm (which is seen in the elder generation) at a younger age by reprogramming genome-wide circadian transcription and the circadian clock to adapt themselves to the changing environment (Kuintzle et al., 2017). A study where changes in lipoperoxidation in the brain and involvement of SOD and glutathione in lipoperoxidation between day and night rhythm was done, showed that activity of lipoperoxidation and SOD is more and GPX activity is less in the night than during the day in the brain of a rat. These changes are physiological processes attributed to changes in light during day and night (Díaz-Muñoz et al., 1985). A study to understand the role of the circadian clock, regulation of GSH rhythm and its production, and the expression and activity of GCL in flies, stated that in the flies with non-mutated clock genes, the activity of GSH and GCL was in rhythm whereas in mutants this was not evident. These parameters being elevated round the clock increased the production of GSH and resulted in an imbalance of redox homeostasis leading to oxidative damage (Beaver et al., 2012). When low molecular weight antioxidant N-acetyl-l-cysteine was supplemented to the BMAL1 deficient mice, the aging process was ameliorated which suggested that aging was due to increased ROS in mice that was decreased by supplementing the antioxidant. Also, the promoter regions of the genes like *Sod-1, GPx-1, Cat*, show the E-box elements implying that these genes are regulated by *BMAL1:CLOCK*complex transcriptionally (Kondratov et al., 2009). A study that checked whether cancer can alter the circadian rhythm and assessed the impact of melatonin on the antioxidant levels in tumors of the liver induced by nitrosodiethylamine in *Mus booduga* mouse, showed that there was a disruption of the circadian clock in the mouse and these effects could be reversed by melatonin (Verma et al., 2014). Circadian rhythm is an emerging field of research in cancer biology as its disruption predisposes to the onset of several chronic diseases including cancer by dysregulating the cell cycle.

## The Peripheral Clock and the Cell Cycle

The peripheral clock is tissue-specific and differs in different tissues bringing about rhythmicity to many physiological processes. As mentioned earlier, the clock and the clock-related genes expressed in all the metabolizing cells of our body are being controlled by an autoregulatory TTFL. The cell cycle which is another oscillatory system is tightly coupled with the circadian rhythm and shares several features to drive their oscillatory events. The clock genes in turn modulate several transcription factors like cyclin B1-Cdc2 kinase which is the crucial regulator of mitosis and controls various tumor suppressor genes and cell-cycle-related genes (Matsuo et al., 2003). Within the cyclin B-Cyclin Dependent Kinase (CDK) complex, the regulatory subunits comprising the cyclins are oscillating while the catalytic subunits comprising the CDKs are present at constant levels. Cell cycle checkpoints tightly regulate the entry of the cells into different phases of the cell cycle. There are two important checkpoints; one in the late G1 phase beyond which the cell can complete the cell division autonomously, and another is the G2/M checkpoint which ensures the stability of the genome before it enters the M phase (Farshadi et al., 2020). DNA damage response pathways are stimulated when the cells are subjected to genotoxic agents and pause the cell in the G2 phase *via* modulating the regulators of the cyclin B-CDK complex.

**FIGURE 3:**
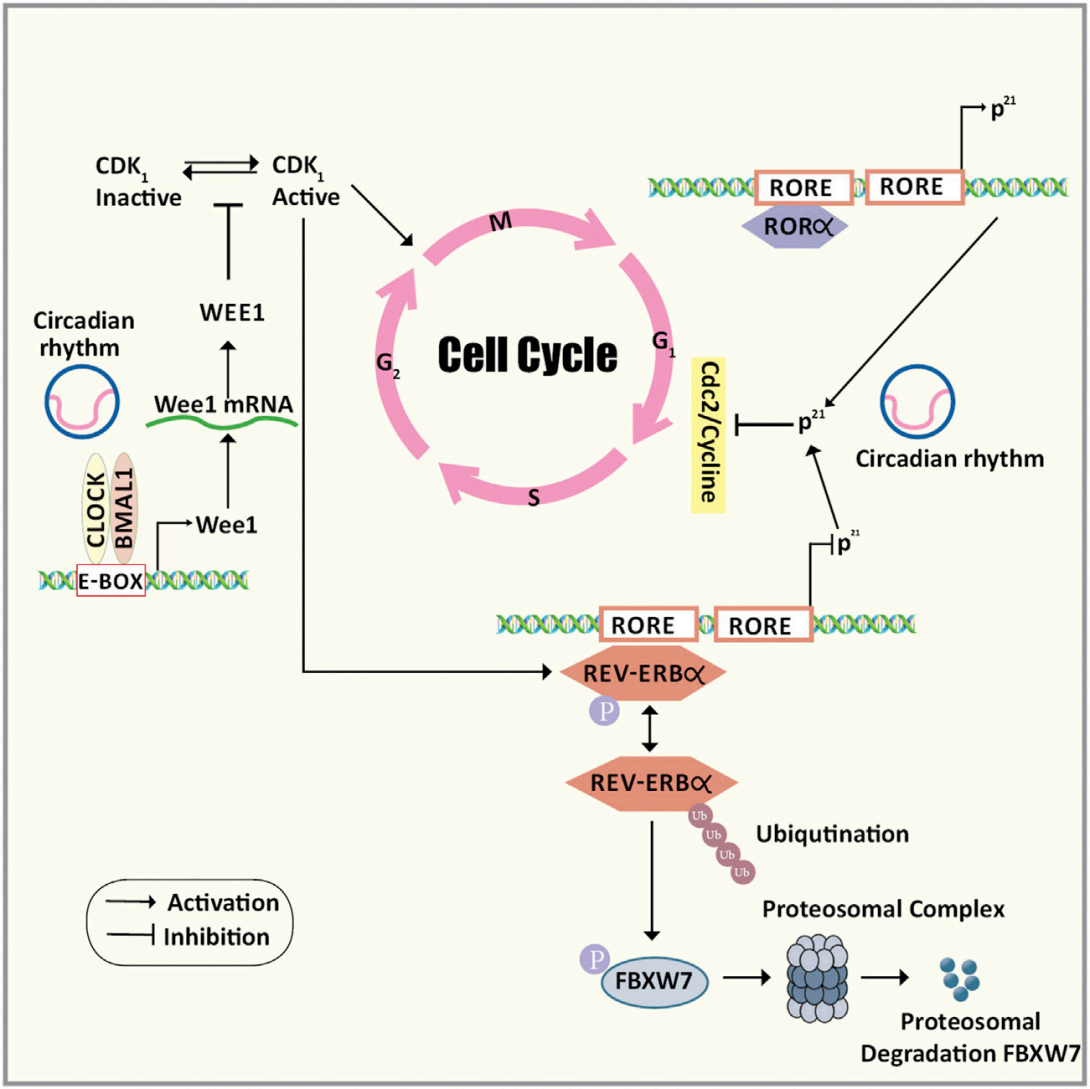
The cross-talk between the cell cycle and the circadian rhythm. The cross-talk between the cell cycle and the circadian rhythm can occur via three major molecular mechanisms. Firstly, mitosis inhibitor protein kinase WEE1 regulates the G2/M transition by phosphorylating the amino acids of Cdk1 thereby inhibiting the kinase activity and preventing entry into mitosis. Wee1 gene promoter consists of CLOCK/BMAL1 responsive E-box elements that control the expression of WEE1 in a circadian manner. Secondly, the transition of the G1 to S phase is under the circadian control ROR/REV-ERB pathway by targeting the p21Waf1/CIP1 a cyclin-dependent kinase inhibitor that negatively regulates the cell cycle progression. Thirdly, the phosphorylation of REV-ERBα via CDK1 targets this clock transcription inhibitory component for ubiquitination and degradation by the F-box protein FBXW7. Abbreviations: BMAL1, Bone Muscle Arnt-like protein 1; CDK1, Cyclin-dependent kinase 1; CLOCK, Circadian Locomotor Output Cycles Kaput; E-box, Enhancer box; F/box/FBXW7, F-Box And WD Repeat Domain Containing 7; G1 phase, Gap1 phase; G2/M transition, Gap2/Mitosis transition; p21Waf1/CIP1, Cyclin-dependent kinase inhibitor 1; REV-ERBα, nuclear receptor subfamily 1, group D, member 1; ROR, Retinoic acid receptor-related Orphan Receptor; S phase, Synthetic phase.

The cross-talk between the cell cycle and the circadian rhythm can occur via three major molecular mechanisms (Figure 3). Firstly, mitosis inhibitor protein kinase WEE1 regulates the G2/M transition by phosphorylating the amino acids of Cdk1 thereby inhibiting the kinase activity and preventing entry into mitosis (Lévi et al., 2007). *Wee1* gene promoter consists of CLOCK/BMAL1 responsive E-box elements that control the expression of WEE1 in a circadian manner. Secondly, the transition of the G1 to S phase is under the circadian control ROR/REV-ERB pathway by targeting the p21^Waf1/CIP1^ a cyclin-dependent kinase inhibitor that negatively regulates the cell cycle progression (Gréchez-Cassiau et al., 2008). Thirdly, the phosphorylation of REV-ERBα via CDK1 targets this clock transcription inhibitory component for ubiquitination and degradation by the F-box protein FBXW7 (Zhao et al., 2016). Also, the circadian clock proteins regulate the DNA damage checkpoints by p53 phosphorylation and stabilization by PER2. This enhances the transcription of p53 target genes like p21 (Gotoh et al., 2014). The above evidence supports the hypothesis that there exists a well-coordinated regulatory mechanism between the two cyclic events and they also share common regulators. Any disturbances in the key regulatory circuitry component of the circadian clock can lead to clock disruption resulting in uncontrolled cell division and cancer.

## Oxidative Stress-Induced Cell Cycle Deregulation

Cyclin-D, which plays a crucial role in initiating the cell cycle in association with Cdk4 or Cdk6 is sensitive to various extracellular stimuli including oxidative stress which modifies its activity through post-transcriptional and post-translational modulations (Stacey, 2010). It is typically found in the G1 phase forming complexes with Cyclin A/E and Cdk2 and promotes the transition through the G1/S phase. Under sustained oxidative stress, cyclin D1 via cross-talk with Cdk1 DNA damage checkpoint pathway plays a pivotal role in cell cycle progression at both G1 and G2 phases. This, along with several other reports has shown that oxidative stress rapidly depletes the cyclin-D1 levels and arrests the cells in the G2 phase (Pyo et al., 2013). On exposure to various stress, cyclin-D1 located in the nucleus is phosphorylated and exported to the cytoplasm where it is ubiquitylated and subjected to proteolysis. In addition to the activation of the proteolysis system, eIF2α-mediated translational repression is required for the complete regulation of cyclin-D1. PERK directly phosphorylates eIF2α under oxidative stress conditions and is responsible for cyclin D1 depletion (Pyo et al., 2013). Nrf2 is a PERK substrate and its nuclear translocation occurs in a PERK-dependent manner following endoplasmic reticulum stress. This suggests that Nrf2 plays an essential role in down-regulating cyclin D1 and promoting cell cycle arrest at excessive levels of oxidative stress (Márton et al., 2018).

Nrf2 may act as a proto-oncogene-inducing cell cycle deregulation because of oxidative stress. The cell proliferation and cell cycle progression also require Nrf2 dependent GSH induced redox signaling (Reddy et al., 2008). In Nrf^−/−^ cells, deregulation of the growth factor-induced ERK1/2, Stat3, and Akt kinase signaling is observed. Deficiency of Nrf2 results in decreased levels of GSH affecting the expression of certain genes due to the activation of transcription factors by the dysregulated signal transduction pathways. Growth arrest is predominantly seen in the G2/M phase following the genetic disruption of Nrf2 with a relatively low cell number in the S phase. As most complex diseases generate oxidative stress in the human cellular system exploring the role of Nrf2 in controlling cell cycle progression is very essential.

## Nrf2 Modulation in Breast Cancer

Nrf2 like in any other cancer, exhibit a dual role in breast cancer as well. Both antioncogenic and prooncogenic activities of Nrf2 are observed based on several additional factors which need to be explored thoroughly. The cell proliferation and migration were significantly reduced in the MDA-MB-231 and MCF-7 breast cancer cells upon Nrf2 genetic silencing by downregulating RhoA (Zhang et al., 2016). The promoter region of the estrogen-related receptor α (ERR1) has a binding region for Nrf2, upon binding it represses the transcription of ERR1 which in turn results in the stabilization of RhoA thus facilitating pro-oncogenic signals. This partly explains the poor prognosis in breast cancer patients with increased Nrf2 expression. In contrast to this, few studies suggest that decreased Nrf2 signaling promotes cell proliferation and cancer growth. The cell division cycle-dissociated protein 4 (CDCA4) knock-down by RNA interference in MCF-7 human breast cancer cell lines resistant to Adriamycin (MCF-7/ADM) decreased the percentage of cancer cells by 50%. The apoptotic rate of the cells significantly increased suggesting that CDCA4 increased the proliferation of cancer cells in MCF-7/ADM human breast cancer cells. CDCA4 is a potential downstream target gene regulated by Nrf2 and is shown to be upregulated by the genetic silencing of Nrf2 (Xu et al., 2018).

The role of Nrf2 in the invasion, migration, and metastasis of breast cancer is still a matter of intense debate as the available literature has conflicting results. A flavonoid found in various food, fisetin, reduced cell migration and colony formation in 4T1 and JC breast cancer cells by decreasing the enzyme activities of matrix metalloproteinases (MMP-2 and MMP-9). The Nrf2 expression in the nuclear fraction was increased by fisetin which also increased the mRNA and protein expression of HO-1 in turn decreasing the activity of MMP-2 and MMP-9. These changes were reversed by Nrf2 silencing suggesting that the metastatic potential of breast cancer can be attenuated by Nrf2 dependent activation of HO-1 (Tsai et al., 2018). In contrast to this, several studies point towards the pro-oncogenic role of Nrf2 promoting tumor invasion and metastasis in breast cancer. For example, Nrf2 expression positively correlated to the expression of oncoprotein mammalian hepatitis B X-interacting protein (HBXIP) in breast cancer tissue suggesting their role in its development. The ROS levels were reduced by HBXIP in the MCF-7 breast cancer cells by promoting the nuclear translocation of Nrf2 and activating the target genes (Zhou et al., 2019). Thus two different mechanisms may be employed for cancer therapy targeting the Nrf2 pathway in breast cancer 1. Activation of Nrf2 to prevent cancer development 2. Inhibition of Nrf2 to improve sensitivity to cancer therapy.

## Small Molecules Targeting Nrf2 in Breast Cancer

After understanding the link between the circadian clock, oxidative damage, and cancer, Nrf2 may be considered as the novel therapeutic target to treat cancer. Several works are underway to develop small molecule-based inhibitors of Keap1/Nrf2 using several approaches like target-based virtual screening, fragment-based drug design, high-throughput screening, structure-based optimization, etc (Zhuang et al., 2017; Rahman et al., 2020). Different compounds which inhibit the Nrf2 pathway are developed and used in the treatment of cancers that act by blocking the synthesis of a protein or decreasing the gene expression and activity of proteasomes or suppressing oxidative stress to inhibit the nucleus translocation of Nrf2 or inhibiting Nrf2-DNA binding (Lin et al., 2020). The natural molecules also play a vital role in targeting breast cancer via the Nrf2 pathway. Curcumin, a polyphenol is one such metabolite, which prevents cancer by Nrf2 signaling activation, modulation of inducible nitric oxide synthase (iNOS) and cyclo-oxygenase (COX2), restoring tumor suppressor p53, and inducing phase-2 antioxidant enzymes glutathione-S-transferase (GST), glutathione reductase (GR), and NQO1 (Das and Vinayak, 2015). Another study has shown that curcumin induces heme oxygenase-1 protein levels and Nrf2 in a dose-dependent manner in HBL100 and MDA-MB-468 breast cancer cell lines. Further, they showed that curcumin induced heme oxygenase-1 and Nrf2 in wild-type mouse embryo fibroblasts (Andreadi et al., 2006). Jain et al. examined the interaction of Amino-1-methyl-6-phenylimidazo [4,5-b]pyridine (PhIP), a known carcinogen found in cooked meat with curcumin in breast epithelial cells, and stated that curcumin inhibited DNA adduct formation, DNA double-strand breaks, and formation of ROS induced by PhIPin normal breast epithelial cells (MCF-10A). They concluded that activation of Nrf2 reduces the DNA adduct formation and oxidative stress through signaling pathways like the antioxidant pathway, caspase activation pathway, and tumor suppressor pathway (Jain et al., 2015). Curcumin also inhibited the proliferation of MCF-7 breast cancer cells through Nrf2-mediated down-regulation of DNA repair-specific nuclease, Flap endonuclease 1 (Fen1)expression by reducing the Nrf2 recruitment to the Fen1 promoter (Chen et al., 2014). Resveratrol, a polyphenol seen in grapefruit, regulate estrogen homeostasis based on NRF2-UGT1A8 (UDP-glucuronosyltransferase 1A8-phase II drug-metabolizing enzymes involved in catechol estrogens metabolism) signaling pathway in MCF-10A. The expression of mammary NRF2–UGT1A8 is down-regulated in breast cancer rats, and treatment with resveratrol upregulates the expression of NRF2–UGT1A8, increases the elimination of catechol estrogens, halts DNA damage induced by estrogen, and reduces breast cancer development, which was abolished by small-interfering RNA-mediated silencing of NRF2. Further by luciferase reporter assay, it was shown that resveratrol triggered the UGT1A8 expression by up-regulating the NRF2 transcriptional activity (Zhou et al., 2018). Female August Copenhagen Irish rats treated with Resveratrol, 17β-estradiol (E2), and resveratrol with E2 showed an upregulation of NRF2 expression in mammary tissues along with increased expression of antioxidant genes NQO1, SOD3, and OGG1in resveratrol alone and resveratrol with E2 treated rats. Further small-interfering RNA-mediated silencing of NRF2 showed inhibition of resveratrol mediated protective effects on the formation of the mammosphere and the colony. Hence concluded that resveratrol inhibits breast carcinogenesis induced by E2 through the NRF2 pathway (Singh et al., 2014). Epigallocatechin-3-gallate (EGCG), a polyphenol found in green tea increases Nrf2 levels in MCF-7 and MDA-MB-231 breast cancer cell lines making them resistant to the inhibitory effects of paclitaxel and doxorubicin (Hu et al., 2010). The combination of the Nrf 2siRNA and EGCG downregulated the intracellular Nrf2 protein levels and reversed the tamoxifen resistance in tamoxifen-resistant MCF-7 (MCF-7/TAM) cells (Esmaeili, 2015). Brusatol, a quassinoid plant extract from *Bruceajavanica* is a novel inhibitor ofNrf2 that sensitizes HER2-positive cancer cells to trastuzumab both *in vitro* and *in vivo*. This indicates the potential role of brusatol as an adjuvant drug along with trastuzumab in HER2-positive cancers treatment (Telkoparan-Akillilar et al., 2019; Yang et al., 2020). These results suggest that using various strategies like modulating the circadian rhythm genes to inhibit Nrf2 may sensitize the tumor cells to chemotherapeutic drugs.

Only a few compounds targeting Nrf2 activity have been considered for Phase II trials. As over activation of Nrf2 has been linked to cancer progression as well as resistance to cancer chemotherapy, Nrf2 inhibitors may be useful in improving the efficacy of cancer therapy (Kansanen et al., 2011; Wong et al., 2016). Over a decade, compounds targeting Cysteine 151 of Keap-1 have been considered for Phase II trials. When the protective response mediated by NRF2 is required, this “cysteine-code” regulates KEAP1 activity (Robledinos-Antón et al., 2019). According to the clinicaltrials.gov identifier RTA-408 (NCT02142959), Sulforaphane (NCT00843167) and Sulforadex (NCT02970682) are under clinical development.

Even though many drugs targeting Nrf2 are being tested in breast cancer cell lines and animal models, only three have passed the Phase I trial. Furthermore, there are no drugs that have been approved for clinical trials that restore circadian rhythm in Nrf2 activated breast cancer. Moreover, it is of prime importance to consider the timings of the drug administration to the patients that may reduce the toxicity of the drug as tissue susceptibility to chemotherapeutic drugs are highly dependent on the chronotype of the patient (Montaruli et al., 2021). One possible explanation on why the Nrf2 targeted drug development is moving at a snail’s pace is the potential off-target effects of Nrf2 activators. Our review gives insight into the molecules that target the anti-oxidant pathways and circadian rhythm in breast cancer. However, future studies should consider the possibility of designing the target-specific compounds with a good pharmacokinetic profile.

## Small Molecules Targeting Circadian Rhythm

The research on the treatment of cancer and its prevention is still growing and the emerging approach of developing small molecules targeting the circadian clock may give promising results. A study where small molecule, REV-ERB agonists like SR9011 and SR9009 and were used showed that they could repress certain processes like metabolism, cell proliferation, and inflammation that are required for tumorigenesis (Scheiermann et al., 2013). REV-ERBα controls autophagy which is modulated in a circadian manner. SR9009 and SR9011decreased the number of autophagosomes and accumulation of p62, which is a protein degraded by autophagy in cancer lines. There was also an increase in LAMP1, lysosomal protein indicating that REV-ERB agonists halted lysosome turnover in cancer lines. Hence stated that small molecule, REV-ERB agonists inhibit autophagy in cancer (Sulli et al., 2018). SR9009, REV-ERB agonist is shown to act as a novel therapeutic target to treat small cell lung cancer, suppressing autophagy by inhibitingthe interaction between REV-ERBα and Atg5 which is a downstream autophagy gene target (Shen et al., 2020).

The interaction between BMALI and CLOCK is disrupted by CLK8, CLOCK-binding small molecule, thus hampering the translocation of CLOCK into the nucleus. CKL8 stabilizes the core clock negative arm by reducing the levels of PER2 in the TTFL positive arm and enhances the magnitude of circadian rhythm in cells. Thus CLK8, the circadian rhythm enhancing molecule may act as a new therapeutic for the diseases related to altered circadian rhythm (Doruk et al., 2020). The clock modulating compound, L-methyl selenocysteine (MSC) upregulates the transcription of Bmal1 and prevents toxicity caused by cyclophosphamide, a chemotherapeutic agent (He and Chen, 2016). KS15, the derivative of 2-ethoxypropanoic acid inhibits CRY-mediated feedback on CLOCK/BMAL1 by binding to terminal domains of CRY-C and enhancing the circadian oscillations of *Bmal1* and *Per2* promoter activity (Cha et al., 2019). It would be interesting to know if these small molecules have any role in inhibiting the Nrf2 protein and thereby overcoming the chemoresistance.

Many studies and research are still in progress to connect the temporal axis into the physiology of humans and medicine to provide an opportunity to stabilize the circadian rhythm to the existing environment, thus helping to create a chance to treat the disease by providing better pharmacological interventions.

## Conclusion

Several processes in our body like cell proliferation, inflammation, DNA repair, metabolism, etc occur cyclically and are modulated by the rhythmic activity of the circadian clock. Any chronic disruption of the circadian rhythm, dysregulates these processes, increasing the risk of individuals for tumor development. Disruption of the components of the circadian rhythm is commonly seen in breast cancer suggesting that pharmacological modulation of the circadian machinery can be considered as an effective therapeutic strategy. Altered circadian rhythm leads to increased oxidative stress resulting in the nuclear translocation of Nrf2, where it binds to the ARE initiating the transcription of several cytoprotective genes. However, Nrf2 acts as a double-edged sword having both antioncogenic and prooncogenic activities. Maximum efficacy with minimum side effects for any particular therapy can be achieved if the treatment strategy regulates the circadian rhythm. Though targeting the Nrf2 activity in the malignant cells can be considered as an ideal strategy to overcome cancer growth, an ideal inhibitor should not only be specific with potent efficacy but also have good bioavailability, bioactivity, and less toxicity. Nevertheless, none of the inhibitors tested to date have yielded practicable results probably due to the cross-talks the Nrf2 pathway has with other pathways like circadian rhythm. New novel approaches may be designed targeting both pathways such that the Nrf2 inhibition occurs in a microenvironment that has a stable circadian rhythm.
